# Falls after hip arthroscopy for femoroacetabular impingement syndrome: Prevalence and risk factors for revision hip arthroscopy

**DOI:** 10.1002/jeo2.70887

**Published:** 2026-08-17

**Authors:** Nicholas R. Kossoff, Manuel A. Romero‐Padron, Adam Alakhras, Nihal Nagesh, Joshua S. Everhart

**Affiliations:** ^1^ Department of Orthopaedic Surgery Indiana University School of Medicine Indianapolis Indiana USA; ^2^ Western Michigan University Homer Stryker M.D. School of Medicine Kalamazoo Michigan USA

**Keywords:** capsular insufficiency, femoroacetabular impingement syndrome, hip joint instability, patient‐reported outcome measures, revision surgery

## Abstract

**Purpose:**

The prevalence and clinical consequences of falls in the early post‐operative period after hip arthroscopy for femoroacetabular impingement syndrome (FAIS) remain unknown. The purpose of this study was to determine the prevalence of and risk factors for postoperative falls within 90 days of surgery in patients who undergo hip arthroscopy for FAIS and determine whether falls within 90 days are associated with increased risk of early revision surgery.

**Methods:**

Consecutive patients who underwent primary hip arthroscopy for FAIS were included. Falls were defined by ground impact by body parts other than the feet, and near‐falls were defined as sudden, forceful and unanticipated loading of the operative extremity without impact. Bivariate and multivariate analyses were conducted to identify associations between demographic or clinical factors and fall occurrence as well as revision surgery within 1 year.

**Results:**

A total of 192 patients met inclusion criteria (median age 36.7 years, interquartile range [IQR] 23.4, 46.5; 72% female). Of these, 33 patients (17%) experienced a postoperative fall or near‐fall (21 falls, 12 near‐falls). Median time to fall was 16.5 days (IQR 8.3–44.5). Fall/near‐fall patients had significantly higher rates of revision surgery within 1 year compared to controls (27% vs. 4%, *p* < 0.001; odds ratio [OR] = 9.4, 95% confidence interval [CI] 3.1–30.5). Fall/near‐fall events were associated with worse baseline physical function (Physical Function Short Form of the Hip Disability and Osteoarthritis Outcome Score [HOOS‐PS], *p* = 0.047), lower mental health scores (Mental Health Inventory‐5 [MHI‐5], *p* = 0.04) and lower activity levels (University of California, Los Angeles [UCLA] score, *p* = 0.03). Hearing or visual impairment (OR = 6.6, 95% CI 1.9–23.3, *p* = 0.004) and higher HOOS‐PS score (OR = 1.1, 95% CI 1.1–1.3, *p* = 0.02) were significant predictors of postoperative falls/near‐falls.

**Conclusion:**

Falls and near‐falls within 90 days after hip arthroscopy were associated with higher rates of revision hip arthroscopy. Surgeons should counsel patients, particularly those with sensory impairments, regarding postoperative fall prevention and emphasize prompt reporting of any fall/near‐fall events. Future studies are needed to determine whether reducing postoperative falls can decrease revision rates.

**Level of Evidence:**

Level III.

**Abbreviations**
ADULT SIM: Adult Single Item Measure physical activity
BMI: body mass index
CI: confidence interval
CONSORT: Consolidated Standards of Reporting Trials diagram
FAIS: femoroacetabular impingement syndrome
GED: general educational development
HOOS‐PS: Physical Function Short Form of the Hip Disability and Osteoarthritis Outcome Score
HS: high school
iHOT‐12: international Hip Outcome Tool
IQR: interquartile range
kg: kilogram
m: metre
MHI‐5: Mental Health Inventory‐5
MRI: magnetic resonance imaging
OR: odds ratio
PASS: Patient‐Acceptable Symptoms State
S/P: status post
THA: total hip arthroplasty
UCLA: University of California at Los Angeles

## INTRODUCTION

Femoroacetabular impingement syndrome (FAIS) and acetabular labral tears are a leading cause of hip pain and dysfunction in active adults. FAIS is associated with substantial pain, reduced physical function, diminished quality of life and considerable preoperative morbidity while patients await surgical treatment [6, 21, 24, 27]. Oftentimes, labral tears co‐occur with FAIS and are a major contributor to hip instability and degeneration [6, 21]. Typically, non‐operative management is first line for FAIS with a labral tear; however, persistent symptoms and dysfunction may necessitate surgical intervention [6].

Hip arthroscopy has become the standard surgical intervention for FAIS with concomitant labral tear, aiming to restore hip function, relieve symptoms and delay the progression to osteoarthritis. Although hip arthroscopy reliably improves patient‐reported outcomes and facilitates return to activity, objective restoration of normal hip biomechanics may be incomplete following surgery, suggesting that functional deficits may persist despite symptomatic improvement [10, 11, 14, 24]. However, the procedure is not perfect and can lead to complications including residual hip impingement, scar tissue formation, insufficient labral healing, chondrolabral breakdown and advancement of osteoarthritis [6, 7, 11, 21, 25]. When complications occur, revision hip arthroscopy may be warranted. Current literature suggests that revision rates for hip arthroscopy range from 1.3% to 26.7% within 5 years of primary surgery, with conversion to a total hip arthroplasty (THA) ranging from 0% to 32.5% depending on the patient age, level of joint degeneration and duration of follow‐up [7, 11, 12, 14, 15, 21].

One potentially avoidable cause of revision hip arthroscopy or conversion from primary hip arthroscopy to THA is postoperative falls. Falls are a widely recognized postoperative complication for orthopaedic procedures such as total hip and knee arthroplasties [16, 26]. However, a notable gap exists in the current literature regarding the prevalence of postoperative falls in patients who have undergone hip arthroscopy for FAIS with contaminant labral tear. As no such study identifying the prevalence of postop falls for hip arthroscopy exists, information on fall risk factors and rates of revision hip arthroscopy in this patient population is lacking. Falls are a widely recognized postoperative complication leading to revision surgeries for other orthopaedic procedures, such as THA, with well‐documented rates of revisions [26]. Falls following arthroplasty have been shown to contribute to implant instability, dislocation, periprosthetic fractures and early revision [2, 16, 26], underscoring the potential for similar associations in patients recovering from hip arthroscopy. Given that hip arthroscopy patients are often younger, more active and expected to resume higher levels of physical activity, the implications of postoperative falls in this group may be clinically significant yet underrecognized.

This study aims to (1) determine the prevalence of and risk factors for postoperative falls within 90 days of surgery in patients who undergo hip arthroscopy for FAIS and (2) determine whether falls within 90 days are associated with increased risk of early (within 1 year) revision hip arthroscopy. Our hypothesis is that patients who sustain postoperative falls will have higher rates of revision hip arthroscopies and more fall risk factors.

## METHODS

### Patient selection

After institutional review board approval, patients were retrospectively identified from a single surgeon's outpatient practice, encompassing all individuals who underwent hip arthroscopy for the diagnosis of FAIS and an acetabular labral tear between August 2024 and February 2025. All patients included in this study provided informed consent prior to participation. Inclusion criteria were (1) age 14–95 years, (2) history of hip arthroscopy for a diagnosis of FAIS or acetabular labral tear between August 2024 and February 2025 by the participating surgeon and (3) complete records, including preoperative patient‐reported outcome surveys and postoperative examinations and surgical records, were available. Exclusion criteria were (1) patients who underwent hip arthroscopy for a diagnosis other than FAIS and acetabular labral tear (acetabular dysplasia, femoral head avascular necrosis, etc.) and (2) history of paediatric hip condition treated non‐surgically, including but not limited to hip dysplasia or Legg–Calve–Perthes disease. Additionally, patients with advanced degenerative hip disease, including Tönnis Grade 2–3 osteoarthritis or joint space narrowing less than 2 mm, were not considered candidates for hip arthroscopy in this practice and therefore were not included in the study cohort.

For the purposes of this study, diagnosis of FAIS required both radiographic evidence of impingement morphology and corresponding clinical symptoms. Patients were diagnosed with FAIS based on a combination of imaging findings and a compatible clinical presentation, including activity‐related anterior hip or groin pain reproduced with provocative examination manoeuvres such as flexion, adduction and internal rotation (FADIR) testing. In cases where the source of symptoms was unclear, an ultrasound‐guided intra‐articular anaesthetic injection was utilized to confirm the hip joint as the primary pain generator prior to surgical intervention.

### Data collection

Patient electronic medical records were retrospectively reviewed on Cerner PowerChart (Oracle Health) from the date of surgery to 90 days postoperatively. All notes within this time frame were reviewed for any mention of a fall or near fall. Falls were defined as an unexpected loss of balance resulting in sudden, unanticipated ground‐level impact with body part(s) other than the feet. Near falls were defined as any form of unexpected loss of balance resulting in sudden, unanticipated forceful loading of full body weight onto the operative extremity without ground impact. Only the index hip arthroscopy procedure per patient was included in the study. For patients who underwent surgery on both hips, only the initial (chronologically first) operation was analysed to avoid duplicate inclusion. For patients who experienced multiple postoperative falls, only the first documented fall was included in the analysis to prevent data duplication and minimize bias in overall event rates. Revision hip arthroscopy surgeries were only recorded if the revision procedure was performed within 1 year of the index surgery. All demographic data were self‐reported on the day of surgery. All comorbidities and medical conditions included in the analysis were documented preoperatively and represented baseline patient characteristics present at least 3 months prior to the time of surgery. Postoperative medication management was standardized for all patients. The routine postoperative protocol included opioid analgesics for approximately 3 days, nonsteroidal anti‐inflammatory drugs (NSAIDs) for 3 weeks and muscle relaxants for 3 weeks. Medication utilization beyond the standard postoperative protocol was not systematically recorded.

### Preoperative patient‐reported outcome measures (PROMs)

On the day of surgery, each patient independently completed a structured preoperative survey composed of several validated instruments: the Mental Health Inventory‐5 (MHI‐5), the 12‐item International Hip Outcome Tool (iHOT‐12), the University of California, Los Angeles (UCLA) Activity Score and the Hip Disability and Osteoarthritis Outcome Score‐Physical Function Short form (HOOS‐PS). The iHOT‐12 includes 12 items targeting hip‐related quality of life and responsiveness to clinical interventions [22]. While the iHOT‐12 produces good measures of hip function and symptoms for higher‐level activities, the HOOS‐PS assesses hip‐related function and symptoms associated with lower‐intensity activities and osteoarthritis, demonstrating acceptable reliability, validity and responsiveness [5, 29]. The MHI‐5, a brief five‐item tool, was used to assess both positive and negative mental health states, including indicators of depression and anxiety [4]. Responses are scored from 0 (indicating poor mental health) to 100 (indicating optimal mental well‐being) [4, 32]. To evaluate physical activity levels, the UCLA Activity Score was utilized, which spans a continuum from complete physical inactivity to regular participation in high‐impact sports [33].

### Statistical analysis

Statistical analysis was performed using a standard software package (JMP 17.2; SAS Institute). A meaningful power analysis could not be performed a priori due to lack of previously reported data on rates of falls after hip arthroscopy. However, post hoc analysis determined that an adequate number of fall or near‐fall events were included to identify up to 6 independent risk factors for falls without overfitting a multivariate model. Descriptive statistics were generated for the entire sample and with stratification by study group. Bivariate analyses of categorical data between groups were performed with use of Pearson *χ* ^2^ test or Fisher's exact test as appropriate in the case of low (<5) cell counts. Bivariate analyses of continuous variables between groups were performed using the Wilcoxon two‐sample test after confirming non‐normal distribution of all continuous variables with the Kolmogorov–Smirnov goodness‐of‐fit testing. Multivariate analysis was performed using nominal logistic regression to identify independent predictors of postoperative falls. Variables with clinical relevance or a bivariate association of *p* < 0.10 were entered into the model, and adjusted odds ratios (ORs) with 95% confidence intervals (CIs) were reported. Demographic variables including age, sex, body mass index (BMI) and tobacco use were retained as covariates in the final model to control for potential confounding. Statistical significance was defined as *p* < 0.05. A separate multivariable logistic regression analysis was performed to identify independent predictors of revision hip arthroscopy within 1 year of the index procedure. Variables selected for inclusion were based on clinical relevance and factors associated with falls or revision surgery on bivariate analysis. Adjusted ORs with 95% CIs were reported.

## RESULTS

### Demographics

**Figure 1:**
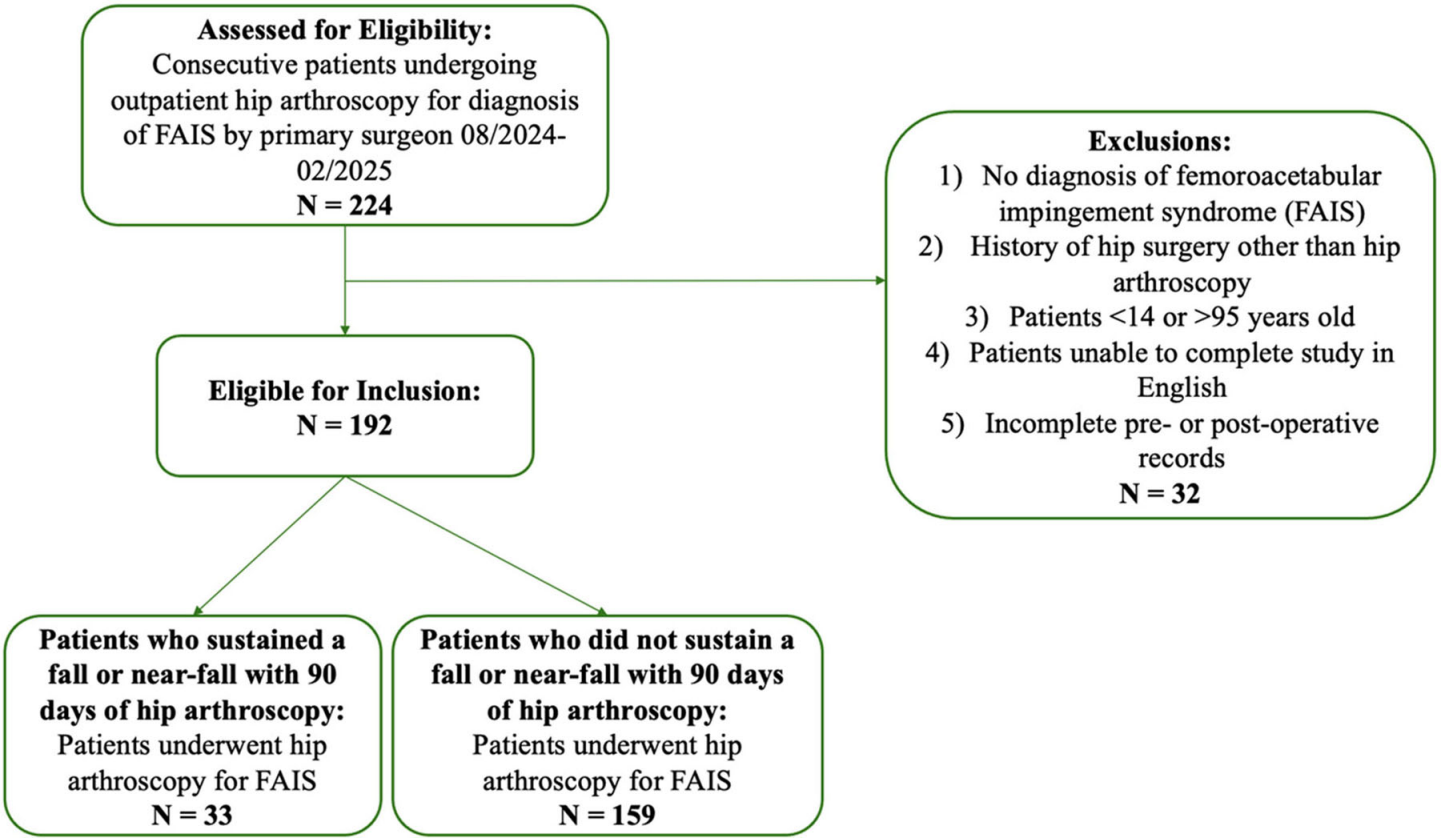
Flow diagram of patient screening, eligibility assessment, exclusions and final study inclusion.

Of 224 consecutive primary hip arthroscopies, a total of 192 patients met inclusion criteria and were included in the current study (Figure 1). The median age of the study cohort was 36.7 years (interquartile range [IQR] 23.4–46.5, range 14.3–74.4 years). In total, 32 patients were excluded: 27 for a diagnosis of acetabular dysplasia in addition to FAIS and/or labral tear, two patients were excluded for femoral head avascular necrosis, and three patients were excluded due to an additional procedure of femoral head core decompression during their arthroscopy. Median age was similar between the fall and near fall groups and the no‐fall group (38.6 vs. 36.7 years, *p* = 0.85) (Table 1). Of the included patients, 21 (11%) experienced a fall with ground impact while 12 (6%) experienced a near fall resulting in sudden and forceful application of full body weight onto the operative leg within 90 days following hip arthroscopy for FAIS. No baseline demographic variables were found to be different when comparing patients who sustained a postoperative fall with ground‐level impact or near fall to patients who did not sustain a postoperative fall (*p* > 0.05).

**Table 1:** Baseline patient demographics.

Variable	Total cohort, *n* = 192	Fall and near‐fall patients, *n* = 33	No‐fall patients, *n* = 159	*p* value
Age (years)	36.7 (23.4, 46.5) (median, IQR)	38.6 (25.9, 45.5) (median, IQR)	36.7 (23.3, 47.1) (median, IQR)	0.85
Sex (*n*)				
Male	53 (28%)	8 (24%)	45 (28%)	0.64
Female	139 (72%)	25 (76%)	114 (72%)	
Race (*n*)				0.88
White	175 (91%)	30 (91%)	145 (91%)	
African American	12 (6%)	2 (6%)	10 (6%)	
Asian or Pacific Islander	4 (2%)	1 (3%)	3 (2%)	
Other	1 (1%)	0 (0.0%)	1 (1%)	
Body mass index (kg/m^2^)	27.4 (23.6, 32.0) (median, IQR)	28.2 (24.5, 35.4) (median, IQR)	27.4 (23.4, 31.2) (median, IQR)	0.27
Tobacco use status (*n*)				0.59
Current user	26 (14%)	6 (18%)	20 (13%)	
Former user	22 (11%)	5 (15%)	17 (11%)	
Never user	141 (73%)	22 (67%)	119 (75%)	
Unknown	3 (2%)	0 (0%)	3 (2%)	
Education level (*n*)				0.71
Some HS or lower	27 (14%)	4 (12%)	23 (14%)	
HS graduate/GED	35 (18%)	6 (18%)	29 (18%)	
Associates/Trade diploma	63 (33%)	12 (36%)	51 (32%)	
Or some college	48 (25%)	7 (21%)	41 (26%)	
Bachelor's degree	13 (7.0%)	4 (12%)	9 (6%)	
Master's degree or higher unknown	6 (3.0%)	0 (0.0%)	6 (4%)	
Employment status (*n*)				0.25
Full time	90 (47%)	15 (45%)	75 (47%)	
Part time	17 (9%)	1 (3%)	16 (10%)	
Unemployed	18 (9%)	5 (15%)	13 (8%)	
Unemployed (disabled)	21 (11%)	4 (12%)	17 (11%)	
Unemployed (student)	35 (18%)	4 (12%)	31 (20%)	
Retired	7 (4%)	3 (9%)	4 (3%)	
Unknown	4 (2%)	1 (3%)	3 (2%)	
Surgery type (*n*)				0.93
Primary hip arthroscopy	162 (84%)	28 (85%)	134 (84%)	
Revision hip arthroscopy	30 (16%)	5 (15%)	25 (16%)	
Surgical side (*n*)				0.56
Left	96 (50.0%)	15 (45%)	81 (51%)	
Right	96 (50.0%)	18 (55%)	78 (49%)	

Abbreviations: GED, general educational development; HS, high school; IQR, interquartile range; kg, kilogram; m, metre.

### Characteristics of falls/Near falls and reporting patterns

Among the 33 (17%) patients who experienced a fall or near‐fall, the median time to fall was 16.5 days (IQR 8.3–44.5) for true falls with ground level impact and 21.5 (IQR 10.0–39.5) for near falls (*p *= 0.94) (Table 2). The majority of events occurred within the first 30 days postoperatively (62% of falls and 67% of near falls). Falls occurred most frequently at home (24%), with other common locations being the stairs (14%), during crutch use (19%) and in the shower (5%). However, the location of falls was unreported in 29% of cases. When comparing those who fell and those who experienced a near fall, the mechanism of reporting differed significantly between groups. Those who fell were more likely to report the event directly to the orthopaedic team via an orthopaedic office visit or an orthopaedic phone note (71% falls vs. 38% near falls, *p* = 0.047), while near falls were more often reported to the patient's physical therapist or another provider (29% falls vs. 62% near falls, *p* = 0.047). No significant differences were found for repeat imaging, emergency department visits, rates of revision hip arthroscopy or physical therapy attendance between fall and near‐fall patients.

**Table 2:** Bivariate analysis of falls versus near falls.

Variable	Fall patients, *n* = 21	Near‐fall patients, *n* = 12	*p* value
Timing of fall (days since surgery)	16.5 (8.3, 44.5); median (IQR)	21.5 (10.0, 39.5); median (IQR)	0.94
Timing of fall (*n*)			1.00
0–30 days	13 (62%)	8 (67%)	
31–60 days	6 (29%)	3 (25%)	
61–90 days	2 (10%)	1 (8%)	
Location of fall (*n*)			0.81
Home	5 (24%)	3 (25%)	
Stairs	3 (14%)	2 (17%)	
Shower	1 (5%)	2 (17%)	
Crutches	4 (19%)	1 (8%)	
Walking	0 (0%)	1 (8%)	
Ice	1 (5%)	1 (8%)	
Car	1 (5%)	0 (0%)	
Unreported	6 (29%)	2 (17%)	
*How fall was reported (n)*			**0.047**
Reported to surgeon/office			
Orthopaedic note	9 (38%)	2 (15%)	
Phone note	8 (33%)	3 (23%)	
Reported elsewhere			
Emergency department	2 (8%)	0 (0%)	
Primary care provider	1 (4%)	0 (0%)	
Physical therapist	4 (17%)	8 (62%)	
Repeat imaging (*n*)			0.47
None	10 (48%)	10 (83%)	
X‐ray	2 (10%)	0 (0%)	
MRI	4 (19%)	1 (8%)	
X‐ray and MRI	4 (19%)	1 (8%)	
Unreported	1 (5%)	0 (0%)	
Emergency department visit (*n*)			0.05
No	12 (57%)	11 (92%)	
Yes	9 (43%)	1 (8%)	
Revision hip arthroscopy s/p fall (*n*)			0.69
No	16 (76%)	8 (67%)	
Yes	5 (24%)	4 (33%)	
Physical therapy attendance/Postoperative restriction compliance (*n*)			1.0
Full attendance	0 (0%)	0 (0%)	
Delayed start	17 (81%)	11 (92%)	
Non‐compliant	1 (5%)	0 (0%)	
Unreported	3 (14%)	1 (8%)	

*Note*: Bold numbers represent a *p* value < 0.05.

Abbreviations: IQR, interquartile range; MRI, magnetic resonance imaging; s/p, status post.

**Figure 2:**
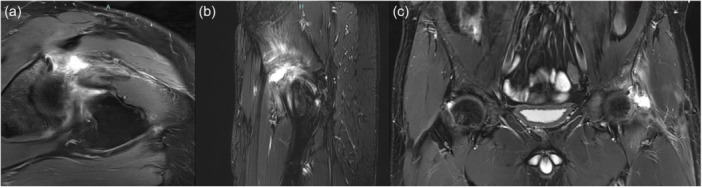
Postoperative magnetic resonance imaging (MRI) of the pelvis demonstrating a capsular defect of the left hip following a postoperative fall within 90 days of left hip arthroscopy for FAIS. (a) Axial, (b) sagittal and (c) coronal sequences show focal discontinuity and signal hyperintensity consistent with a capsular rupture on the left side. Both hips had previously undergone hip arthroscopy, allowing for direct comparison with the contralateral (right) side, which demonstrates intact capsular tissue on the coronal image. FAIS, femoroacetabular impingement syndrome.

Among patients sustaining falls or near falls who required revision arthroscopy (*n* = 9), the most common causes for revision were persistent/recurrent labral tears (5/9), capsular defects (3/9) (Figure 2) and arthrofibrosis (2/9).

### Bivariate analysis of falls and near falls versus no‐falls

Patients who experienced a fall or near fall had a significantly higher rate of revision hip arthroscopy compared to patients who did not fall (27% vs. 4%, *p *< 0.001) (Table 3). The odds of undergoing revision arthroscopy were 9.4 times higher for patients who reported a postoperative fall or near‐fall compared to those who did not (OR = 9.4, 95% CI 3.1–30.5, *p* < 0.001). While compliance with physical therapy and postoperative restrictions showed a trend toward lower adherence in fall/near fall patients, this did not reach statistical significance (*p* = 0.13).

**Table 3:** Bivariate analysis of falls and near falls versus no‐falls.

Variable	Fall and near‐fall patients	No‐fall patients	*p* value
	*n* = 33	*n* = 159	
Age (years) (median IQR)	38.6 (25.9, 45.5) (median, IQR)	36.7 (23.3, 47.1) (median, IQR)	0.85
Sex (*n*)			0.64
Male	8 (24%)	45 (28%)	
Female	25 (76%)	114 (72%)	
Body mass index (kg/m^2^) (median IQR)	28.2 (24.5, 35.4)	27.4 (23.4, 31.2)	0.27
Revision hip arthroscopy (*n*)			**<0.001**
No	24 (73%)	151 (96%)	
Yes	9 (27%)	6 (4%)	
iHOT‐12 (median IQR)	31.7 (20.8, 44.6)	37.5 (24.2, 53.3)	0.16
HOOS‐PS (median IQR)	16 (14, 19)	15 (12, 18)	**0.047**
MHI‐5 (median IQR)	76 (58, 84)	80 (68, 88)	**0.04**
UCLA Activity Scale (median IQR)	3 (3, 4.5)	4 (3, 6)	**0.03**
Physical therapy attendance/Postoperative restriction compliance (*n*)			0.13
Full attendance	28 (85%)	147 (92%)	
Delayed start	1 (3%)	0 (0%)	
Non‐compliant	4 (12%)	10 (6%)	
Unreported	0 (0%)	2 (1%)	
Opioid dependence (*n*)			0.53
No	32 (97%)	156 (98%)	
Yes	1 (3%)	3 (2%)	
Alcohol dependence (*n*)			0.43
No	32 (97%)	157 (99%)	
Yes	1 (3%)	2 (1%)	
Orthostatic hypotension (*n*)			0.06
No	30 (91%)	156 (98%)	
Yes	3 (9%)	3 (2%)	
Hearing/visual impairment (*n*)			**0.002**
No	27 (82%)	153 (96%)	
Yes	6 (18%)	6 (4%)	
Inflammatory arthritis (*n*)			0.21
No	31 (94%)	155 (98%)	
Yes	2 (6%)	3 (2%)	
History of movement disorder (*n*)			0.08
No	27 (82%)	146 (92%)	
Yes	6 (18%)	13 (8%)	
History of skeletal/muscular/nervous system disorder (*n*)			0.78
No	28 (85%)	138 (87%)	
Yes	5 (15%)	21 (13%)	
History of endocrine disorder (*n*)			1
No	29 (88%)	140 (88%)	
Yes	4 (12%)	19 (12%)	
History of migraine (*n*)			0.16
No	28 (85%)	147 (92%)	
Yes	5 (15%0	12 (8%)	
History of blood disorder (*n*)			1
No	32 (97%)	154 (97%)	
Yes	1 (3%)	5 (3%)	

*Note*: Bold numbers represent a *p* value < 0.05.

Abbreviations: HOOS‐PS, the Hip Disability and Osteoarthritis Outcome Score‐Physical Function Short form; iHOT‐12, 12‐item International Hip Outcome Tool (iHOT‐12); IQR, interquartile range; kg, kilogram; m, metres; MHI‐5, Mental Health Inventory‐5; UCLA, University of California, Los Angeles.

Several preoperative PROMs were associated with postoperative fall or near‐fall events. Patients who experienced a fall or near‐fall had significantly lower preoperative scores on the MHI‐5 (median 76 vs. 80, *p* = 0.04), lower UCLA Activity Scale scores (median 3 vs. 4, *p* = 0.03) and higher HOOS‐PS scores (median 16 vs. 15, *p* = 0.047), indicating worse physical function and mental health status prior to surgery. There was no significant difference in iHOT‐12 scores (*p* = 0.16).

Among the comorbid conditions analysed, hearing or visual impairment was the only factor found to be significantly associated with falls, occurring more frequently in the fall/near‐fall group than in those without falls (18% vs. 4%, *p* = 0.002). There were also non‐significant trends toward higher rates of movement disorders (18% vs. 8%, *p* = 0.08) and orthostatic hypotension (9% vs. 2%, *p* = 0.06) in patients who experienced falls or near falls. However, no other comorbidities showed a significant association with fall events (all *p* > 0.05).

### Multivariate analysis—Independent predictors of falls

After controlling for potential confounders, hearing or visual impairment and HOOS‐PS score remained a significant independent predictor of postoperative falls or near‐falls. Patients with sensory impairments demonstrated a 6.6‐fold increased odds of a fall event compared to those without (OR = 6.6; 95% CI 1.9–23.3, *p *= 0.004) (Table 4). Additionally, worse physical function, as indicated by higher HOOS‐PS scores, was independently associated with greater fall risk (OR = 1.1; 95% CI 1.0–1.3, *p* = 0.02).

**Table 4:** Independent predictors of postoperative falls.

Variable	Odds ratio [95% confidence interval]	*p* value
Hearing/visual impairment (yes)	6.6 [1.9, 23.3]	**0.004**
HOOS‐PS	1.1 [1.0, 1.3]	**0.02**

*Note*: Bold numbers represent a *p* value < 0.05.

Abbreviation: HOOS‐PS, the Hip Disability and Osteoarthritis Outcome Score‐Physical Function Short form.

### Multivariate analysis—Independent predictors of revision hip arthroscopy

After controlling for potential confounders including hearing/visual impairment, preoperative mental health (MHI‐5), physical function (HOOS‐PS) and activity level (UCLA Activity Scale), postoperative fall or near‐fall within 90 days of primary surgery remained an independent predictor of revision surgery within 1 year. Patients who sustained postoperative falls or near falls demonstrated a 9.5‐fold increased odds of requiring revision hip arthroscopy compared to those who did not fall (OR 9.5; 95% CI 2.9–33.2, *p *< 0.001) (Table 5). The median age of patients who underwent revision arthroscopy was 42.0 years (IQR 21.0–46.1; range 17.0–60.5 years).

**Table 5:** Independent predictors of revision hip arthroscopy within 1 year.

Variable	Odds ratio [95% confidence interval]	*p* value
Fall/near‐fall within 90 days of primary surgery (yes)	9.5 [2.9, 33.2]	**<0.001**
Hearing/visual impairment (yes)	1.1 [0.1, 5.8]	0.90
MHI‐5	1.0 [1.0, 1.0]	0.71
HOOS‐PS	1.0 [0.8, 1.2]	0.99
UCLA Activity Scale	1.0 [0.7, 1.3]	0.89

*Note*: Bold numbers represent a p value < 0.05.

Abbreviations: HOOS‐PS, the Hip Disability and Osteoarthritis Outcome Score‐Physical Function Short form; MHI‐5, Mental Health Inventory‐5; UCLA, University of California, Los Angeles.

## DISCUSSION

Despite the growing popularity of hip arthroscopy, few studies have explored the prevalence and clinical implications of postoperative falls and near‐falls in this population. Our findings address this gap in the literature, demonstrating that approximately 17% of patients (*n* = 33) reported either a fall or near‐fall within 90 days after hip arthroscopy. Of these patients, 27% required a revision hip arthroscopy, a rate significantly higher than those who did not fall. The odds of requiring revision arthroscopy were over eight times higher in patients who experienced a postoperative fall or near‐fall compared to those who did not fall (OR = 9.4, 95% CI 3.1–30.5, *p* < 0.001). Importantly, patients who experienced ground‐level falls and those with near‐falls had similar clinical outcomes, but reported these events differently. Falls were more often reported to the orthopaedic surgeon's office, while near‐falls were typically disclosed to physical therapists or outside providers.

While no prior study has specifically assessed fall rates in patients undergoing hip arthroscopy for FAIS, the observed 17% fall/near‐fall rate aligns with previously reported fall rates following other orthopaedic procedures. For example, fall rates after THA have been reported between 12% and 25% in the early postoperative period [26]. Similar risks have been noted following total knee arthroplasty (TKA) and shoulder arthroplasty [2, 20, 26, 31]. These findings suggest that falls are not exclusive to weight‐bearing joints or elderly populations but represent a broader issue in orthopaedic recovery. Compared to these procedures, our observed fall rate in hip arthroscopy, which is often performed in younger, more active patients, is unexpectedly high and warrants clinical attention. Persistent alterations in hip mechanics after arthroscopy may partially explain this vulnerability, as previous investigations have demonstrated that improvements in symptoms do not necessarily correspond with normalization of hip joint motion after osteochondroplasty [10].

Although numerous fall risk factors have been proposed for other orthopaedic surgeries, hearing/visual impairment and worse preoperative physical function were the only independent predictors identified in our cohort. These findings are consistent with previous work demonstrating that patients undergoing hip arthroscopy frequently present with substantial preoperative morbidity, reduced function and impaired hip awareness before surgery, characteristics that may contribute to balance deficits and increased susceptibility to postoperative fall events [27]. This suggests that fall risk may be influenced not only by sensory deficits, but also by preoperative functional limitations. These findings are consistent with fall risk literature in patients with FAIS as well as for other surgical and general populations, where FAIS symptoms and sensory impairments contribute to postural instability and spatial misjudgments [8, 18]. Interestingly, age, sex, body mass index (BMI), physical therapy compliance or any other health comorbidities evaluated were not significantly associated with falls in our cohort. This contrasts with existing literature, which suggests a myriad of factors, notably a history of muscular, movement and gait disorders, being associated with heightened fall risk in other lower extremity orthopaedic procedures [1, 8]. This difference may be due to the comparative age and health status of patients undergoing hip arthroscopy, who are generally younger and more active, versus those undergoing other orthopaedic procedures who tend to demonstrate older age and worse baseline health status [8, 19]. This limits the predictive value of health conditions unrelated to hearing or visual impairments in the hip arthroscopy population. Because of this, surgeons should counsel candidates for hip arthroscopy who have hearing or visual impairments that they have a higher risk of a fall event postoperatively and consider worse preoperative physical function as an additional risk factor that may warrant enhanced postoperative support and monitoring.

The association between postoperative falls and near‐falls and revision hip arthroscopy was substantial, with 27% of patients in the fall/near‐fall group requiring revision arthroscopy. This indicates a significant re‐injury risk for patients who sustain a fall event compared to those who do not. Moreover, multivariate analysis confirmed that falls within 90 days were an independent predictor of revision arthroscopy, with a nearly 9.4‐fold increase in odds, highlighting the clinical importance of fall awareness, patient counselling and prompt evaluation following a fall event. All fall and near‐fall events occurred within the first 90 postoperative days, whereas revision hip arthroscopies were assessed within 1 year of the index procedure, establishing that fall events preceded revision arthroscopy. However, the precise temporal relationship between fall events and subsequent symptom progression was not systematically evaluated. Therefore, while postoperative falls may contribute to recurrent pathology or symptom exacerbation, our findings should be interpreted as demonstrating an association rather than a definitive causal mechanism. Most injuries occurred during routine daily activities such as moving around the home, during crutch ambulation or when using stairs and commonly resulted in symptomatic labral re‐tears, capsular dehiscence/defects, arthrofibrosis or aggravation of previously stabilized postoperative pain. These findings are consistent with prior revision hip arthroscopy literature, which has identified recurrent labral pathology, capsular insufficiency, residual impingement and arthrofibrosis among the most common indications for revision surgery [12, 15, 21, 25]. In the present study, recurrent labral pathology and capsular defects represented the most frequent findings among patients undergoing revision hip arthroscopy following a postoperative fall or near‐fall.

Importantly, although near‐falls lacked direct ground impact, they were still associated with similar rates of revision arthroscopy and emergency department visits compared to complete falls with ground impact. While near‐falls do not result in ground impact, they involve sudden, unanticipated loading of the operative extremity with full body weight during a period when patients are instructed to limit weight‐bearing to protect healing tissues. Consequently, the clinically relevant factor may not be ground impact itself, but rather the unexpected transmission of excess force across recently repaired structures. The similar outcomes observed between falls and near‐falls in our cohort support the potential clinical relevance of both event types. Sudden and high‐intensity joint loading has been shown to create rapid force development and high ground reaction forces that are difficult for compromised or healing tissues to absorb safely [3, 17, 30]. Because of this, current clinical guidelines recommend delaying high‐load, explosive or unpredictable activities until late‐stage rehabilitation, when tissue healing, strength and neuromuscular control are adequately restored [17, 23]. For these reasons, surgeons should err on the side of caution by treating falls and near‐falls with similar clinical suspicion to ensure that new injuries or compromised healing do not go undetected after a fall event. Should a fall or near‐fall occur, surgeons should maintain a high index of suspicion for the necessity of a revision hip arthroscopy.

Although postoperative falls were strongly associated with revision arthroscopy, the observational nature of this study precludes conclusions regarding causality. Patients who experienced falls or near‐falls demonstrated worse baseline physical function, lower activity levels and poorer mental health scores, suggesting that falls may represent a marker of patients predisposed to poorer outcomes rather than the sole mechanism leading to revision arthroscopy. To further evaluate this possibility, an additional multivariable model incorporating preoperative physical function (HOOS‐PS), mental health (MHI‐5), activity level (UCLA Activity Scale) and hearing/visual impairment was performed. Postoperative falls remained independently associated with revision hip arthroscopy, whereas these baseline factors were not significant predictors. Previous studies have identified several risk factors for revision hip arthroscopy, including younger age, female sex, elevated BMI, preoperative opioid use, decreased joint space, degenerative changes, residual femoroacetabular impingement, capsular insufficiency and labral pathology [6, 9, 12, 13, 15, 21, 25, 28]. While these factors remain important contributors to postoperative failure, postoperative falls remained independently associated with revision hip arthroscopy after adjustment for measured baseline patient characteristics in the present cohort. Nevertheless, residual confounding from unmeasured factors remains possible. Future studies with larger cohorts and prospective designs are needed to further clarify the relationship between postoperative falls and revision arthroscopy.

The similar clinical outcomes observed between falls and near‐falls underscore the importance of clinician vigilance during postoperative follow‐up. Although near‐falls do not involve ground impact, both event types expose the operative extremity to sudden, unanticipated loading during a vulnerable phase of tissue healing. In our cohort, falls and near‐falls demonstrated comparable rates of revision arthroscopy, repeat imaging, emergency department utilization and postoperative compliance, supporting our decision to evaluate them together. These findings may have important clinical implications, as near‐falls are less likely to be perceived by patients as clinically significant events and therefore may be underreported. Consequently, injuries related to unexpected postoperative loading may go unrecognized if providers focus solely on traditional falls. Falls were more often reported to the orthopaedic surgeon or care team, while near‐falls were frequently reported only to physical therapists, other providers or went undocumented. Given the similar risks associated with both falls and near‐fall events, we recommend that providers specifically educate patients about the consequences of both and emphasize the importance of promptly reporting these events to the surgical team.

## LIMITATIONS

This study is not without limitations. Patients undergoing hip arthroscopy are typically younger and healthier than those undergoing other orthopaedic procedures, which likely accounts for the lower prevalence of traditional fall risk factors such as older age, multiple comorbidities and neuromuscular disorders. While conditions like movement disorders and orthostatic hypotension are known to increase fall risk, their rarity in our cohort may cause this study to be underpowered to detect associations for these variables. Similarly, the relatively small number of fall/near‐fall events and revision hip arthroscopies may have limited statistical power and increased the potential for model overfitting. This study relied on retrospective chart review and self‐reported fall data, which introduces the potential for underreporting or misclassification bias. Additionally, residual confounding may be present, as patients who experienced falls demonstrated differences in baseline functional status, activity level and mental health that may independently influence postoperative outcomes and revision hip arthroscopy risk. Patients may have chosen not to disclose a fall, especially if asymptomatic or may have downplayed events such as near‐falls. Furthermore, non‐standardized reporting methods (e.g., verbal reports to therapists vs. EMR documentation) could lead to variability in documentation accuracy. Reporting bias is possible, as patients with greater postoperative symptoms may have been more likely to report fall or near‐fall events. Therefore, the true prevalence of postoperative falls may be underestimated. Finally, while hearing and visual impairments were significantly associated with falls, these conditions are also rare in the general hip arthroscopy population. Therefore, while statistically significant, they may not account for the majority of fall risk in this group.

In conclusion, falls and near‐falls within 90 days of hip arthroscopy were associated with higher rates of revision hip arthroscopy. Surgeons should counsel patients, particularly those with sensory impairments, regarding postoperative fall prevention and emphasize prompt reporting of any fall or near‐fall events directly to the surgical team. Future studies are needed to determine whether reducing postoperative falls can decrease revision rates.

## AUTHOR CONTRIBUTIONS

*Conceptualization*: Nicholas R. Kossoff, Manuel A. Romero‐Padron and Joshua S. Everhart. *Methodology*: Nicholas R. Kossoff, Manuel A. Romero‐Padron and Joshua S. Everhart. *Formal analysis*: Nicholas R. Kossoff, Adam Alakhras and Nihal Nagesh. *Investigation*: Nicholas R. Kossoff, Adam Alakhras and Nihal Nagesh. *Resources*: Joshua S. Everhart. *Data curation*: Nicholas R. Kossoff, Manuel A. Romero‐Padron and Joshua S. Everhart. *Writing*: Nicholas R. Kossoff. *Writing—review and editing*: All authors. *Visualization*: Nicholas R. Kossoff and Manuel A. Romero‐Padron. *Supervision*: Joshua S. Everhart. *Project administration*: Joshua S. Everhart.

## FUNDING INFORMATION

The authors have no funding to report.

## CONFLICT OF INTEREST STATEMENT

The authors declare no conflicts of interest.

## ETHICS STATEMENT

This study was approved by the Institutional Review Board at the Indiana University School of Medicine (IRB#22175 and IRB#22178). All participants provided written informed consent (minors provided assent with written consent from a parent or legal guardian).

## Data Availability

The data that support the findings of this study are available from the corresponding author upon reasonable request.
